# Transcription Factor STOX1A Promotes Mitotic Entry by Binding to the CCNB1 Promotor

**DOI:** 10.1371/journal.pone.0029769

**Published:** 2012-01-13

**Authors:** Daan van Abel, Omar Abdul-Hamid, Marie van Dijk, Cees B. M. Oudejans

**Affiliations:** Department of Clinical Chemistry, VU University Medical Center, Amsterdam, The Netherlands; University of Saarland Medical School, Germany

## Abstract

**Background:**

In this study we investigated the involvement of the transcription factor STOX1A in the regulation of the cell cycle.

**Methodology/Principal Findings:**

We found that several major cell cycle regulatory genes were differentially expressed upon STOX1A stimulation and knockdown in the neuroblastoma cell line SH-SY5Y. This includes STOX1A dependent differential regulation of cyclin B1 expression, a cyclin which is known to regulate mitotic entry during the cell cycle. The differential regulation of cyclin B1 expression by STOX1A is direct as shown with chromatin immunoprecipitation. Results furthermore suggest that mitotic entry is enhanced through the direct upregulation of cyclin B1 expression effectuated by STOX1A.

**Conclusions:**

In conclusion we hereby show that STOX1A is directly involved in the regulation of the cell cycle.

## Introduction

Mammalian cell division is controlled by the expression of cyclins and activation of their associated cyclin dependent kinases (CDKs). While the CDK components are generally expressed ubiquitously during the cell cycle, expression of cyclins accumulate periodically during distinct phases (G1, S, G2 and M phase) of the cell cycle [1]. In each phase, binding of cyclins with their corresponding CDK forms an active cyclin/CDK complex. In general, G1 to S phase progression is controlled by CDK2 bound to S-phase cyclins [2] (E- and A-type) whereas G2 to M phase is triggered by CDK1 associated with mitotic cyclins [3] (A- and B-type). Active cyclin/cdk complexes can phosphorylate several substrates, which subsequently trigger cell cycle progression [4]–[6]. Many of these cyclin/cdk complex substrates and regulators of the cell cycle machinery itself have been characterized in detail, and recently it was shown to include a group of proteins belonging to the forkhead transcription factors. These transcription factors are characterized by a 100 amino acid DNA-binding motif termed the winged helix domain [7]–[10]. Several studies have confirmed the role of forkhead transcription factors in regulating the transcription of cell cycle regulatory genes during the cell cycle [8]–[10]. Additionally, it has been shown that multiple members of the forkhead transcription factors are regulated by components of the cell cycle itself. These include FOXM1 [8], FOXO1 [9] and FOXK2 [10].

Recently, Storkhead box 1A (STOX1A), a transcription factor structurally and functionally related to the forkhead family of transcription factors [11], [12], has been shown to be expressed abundantly in the brain and found to be upregulated in advanced stages of Late Onset Alzheimer Disease (LOAD, Braak 3–6). Secondly, STOX1A was found to be expressed at the centrosomes of dividing cells [13]. Centrosomes serve as reaction centres for several key regulators of the cell cycle machinery [14], [15], where in particular G2 to M-phase transition is triggered by cyclin B1-CDK1 [16], [17]. Together with the increasing evidence that neurons, generally in a non-dividing state called G0, re-express a multitude of cell-cycle regulators in Alzheimer's disease (AD) [18]–[20], let us to explore the involvement of STOX1A in cell cycle related events. Here we show that in the neuroblastoma SH-SY5Y cell line STOX1A directly regulates the expression of the mitotic cyclin B1. Hereby we show that STOX1A, in addition to other members of the forkhead transcription factors, is directly involved in regulating the cell cycle. Upregulated expression of STOX1A in LOAD therefore potentially influences neuronal cell cycle re-entry.

## Results

### Expression analysis of SH-SY5Y cells stably transfected with STOX1A during distinct phases of the cell cycle

**Figure 1:**
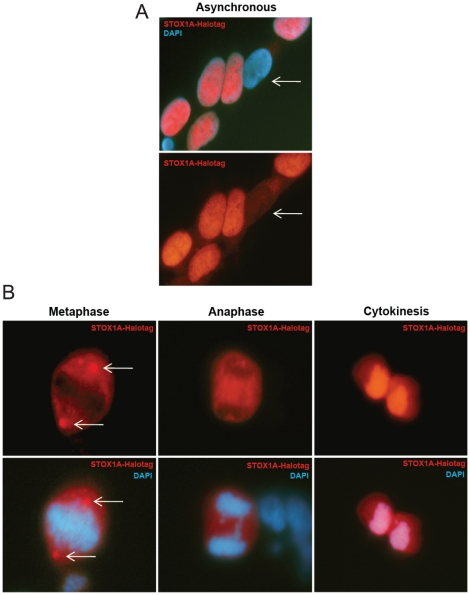
Expression analysis of STOX1A in stably transfected SH-SY5Y cells. (A) Immunofluorescence shows exclusively nuclear or cytoplasmic (white arrows) staining for STOX1A-Halotag protein in STOX1A stably transfected SH-SY5Y cells. (B) Cells undergoing mitosis showing a non-overlapping STOX1A/DAPI immunofluorescence pattern during metaphase and anaphase until cytokinesis when STOX1A immunofluoresence overlaps with DAPI (DNA) immunofluorescence.

To identify the expression pattern of STOX1A in stably transfected SH-SY5Y cells we performed immunofluorescence using an antibody against the Halotag attached to the STOX1A recombinant protein. During interphase we observed primarily nuclear and to a lesser extend cytoplasmic STOX1A staining (Fig. 1A) which confirms the model of STOX1A nucleo-cytoplasmic shuttling as previously described by our lab [12]. Nuclear localization represents the active form of STOX1A.

To investigate the expression pattern of STOX1A during mitosis, cells were arrested at the G2/M-phase boundary. As also observed for the forkhead transcription factor FOXK2 [10], STOX1A shows a non-overlapping immunofluorescence pattern with DNA (STOX1A-halotag/DAPI merge) shortly after nuclear envelope breakdown in prometaphase. The non-overlapping immunofluorescence pattern is best seen during metaphase and anaphase until cytokinesis occurs when STOX1A immunofluorescence overlaps with DNA (DAPI) (Fig. 1B). As shown previously by us [15], STOX1A is concentrated at the centrosomes during metaphase (Fig. 1B, white arrows).

### STOX1A regulates cell proliferation in SH-SY5Y cells

**Figure 2:**
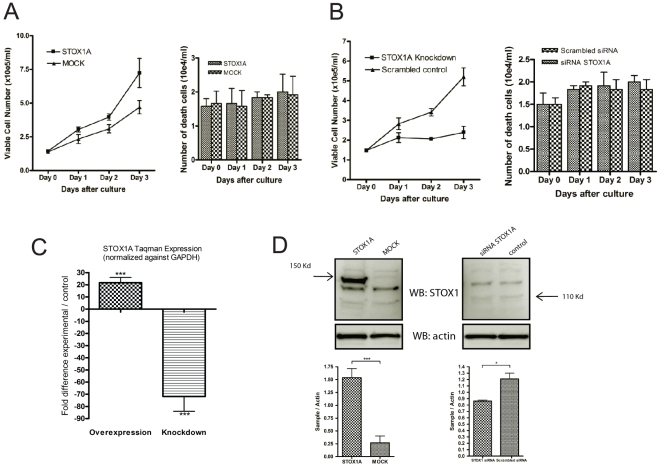
The effect of STOX1A on cell proliferation in the neuroblastoma cell-line SH-SY5Y. (A) The proliferation curve shows significantly decreased cell proliferation for STOX1A siRNA transfected SH-SY5Y cells compared to scrambled controls after 1, 2, and 3 days in culture. (A. left bar) In parallel we did not found significant differences in cell death between STOX1A siRNA and scrambled siRNA transfected cells at each of the indicated time points. (B) The proliferation curve shows significantly increased cell proliferation for STOX1A compared to MOCK stably transfected SH-SY5Y cells after 1, 2, and 3 days in culture. (B. left bar) In parallel we did not found significant differences in cell death between STOX1A and MOCK transfected cells at each of the indicated time points. Bars are mean ± SEM. *P*-values for each timepoint were calculated using two-tailed unpaired *t*-test. (C) Overexpression and knockdown of STOX1A was determined with quantitative RT-PCR showing a mean 21 fold (mean ΔΔCt 4.4) increase in mRNA expression for STOX1A in stably transfected SH-SH5Y cells compared to the controls (Left bar) and a mean 71 fold (mean ΔΔCt 6.15) decreased mRNA expression for STOX1A siRNA transfected SH-SY5Y cells compared to the scrambled controls (right bar). Bars are mean ± SEM. *** indicate *P*<0.001 (one sample t-test with theoretical mean 0). N = 4, each sample was measured in triplicate. (D, Bottom left graph) Quantification of STOX1A-Halotag protein was performed using densitometry. The ratio number of obtained band intensities for STOX1A (at the expected band size of 150 Kd) divided by actin was compared to the ratio number of obtained band intensities for MOCK divided by actin for 3 independent experiments. A significant increase for the STOX1A ratio number was found compared to the MOCK ratio number. (D, Bottom right graph) Quantification of endogenous STOX1A protein was performed using densitometry. The ratio number of obtained band intensities for STOX1A siRNA (at the expected band size of 110 Kd) divided by actin was compared to the ratio number of obtained band intensities for scrambled siRNA divided by actin for 3 independent experiments. A significant increase for the STOX1A siRNA ratio number was found compared to the scrambled siRNA ratio number. *P*-values were calculated using two-tailed unpaired *t*-test, error bars represent ± SEM, * indicate *P*<0.05, *** indicate *P*<0.001. Westernblot images are a representative of at least 3 independent experiments.

As the results above indicate that STOX1A is involved in mitosis, the effect of STOX1A on cell proliferation was tested by using STOX1A siRNA knockdown in comparison to scrambled siRNAs in the neuroblastoma SH-SY5Y cell line. Knockdown of STOX1A resulted in a significant reduction of cell proliferation compared to the scrambled siRNA control (Fig. 2A). In parallel, at each time point, the amount of death cells were counted. However we did not found significant differences in cell death between STOX1A siRNA or scrambled siRNA transfected cells (Fig. 2A, left graph). In concordance with the reduction in cell proliferation upon STOX1A siRNA transfection, cells that were stably transfected with STOX1A showed a significant increase in cell proliferation compared to empty vector (MOCK) transfected SH-SY5Y cells (Fig. 2B). In parallel, at each time point, the amount of death cells were counted. No significant differences in cell death between STOX1A and MOCK transfected cells were found (Fig. 2B, left graph). Knockdown and stable overexpression of STOX1A in the SH-SY5Y cell-line was confirmed at the mRNA (Fig. 2C) and protein level (Fig. 2D).

**Figure 3:**
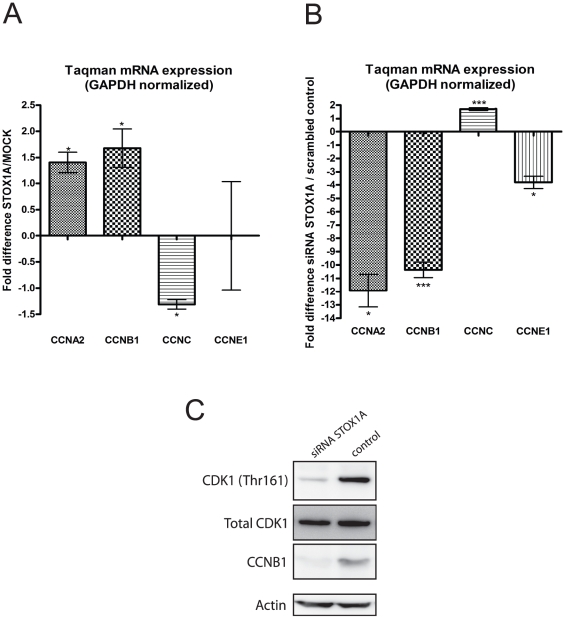
STOX1A induces changes in several major cell cycle regulatory genes. (A) The effect of stable STOX1A overexpression in the SH-SY5Y neuroblastoma cell line on four major mammalian cell cycle regulatory genes was determined with quantitative RT-PCR showing a mean 1,4 fold (mean ΔΔCt is −0,49) and mean 1,72 fold (mean ΔΔCt is −0,78) increased mRNA expression for CCNA2 and CCNB1, respectively, and a mean 1,22 fold (mean ΔΔCt is −0,29) decreased mRNA expression for CCNC in STOX1A stably transfected cell compared to their negative controls. (B) The effect of STOX1A knockdown in the SH-SY5Y neuroblastoma cell line on four major mammalian cell cycle regulatory genes was determined with quantitative RT-PCR showing a mean 11,8 fold (mean ΔΔCt is −3,56), mean 10.3 fold (mean ΔΔCt is −3,37) and mean 3,8 fold (mean ΔΔCt is −1,92) increased mRNA expression for CCNA2, CCNB1 and CCNE1 respectively, and a mean 1,7 fold (mean ΔΔCt is −0,77) increased mRNA expression for CCNC in STOX1A siRNA treated cells compared to their negative controls. (A,B) Bars are mean ± SEM. * indicate *P*<0.05, ** indicate *P*<0.01, *** indicate *P*<0.001 (one sample t-test with theoretical mean 0). N = 4, each sample was measured in triplicate. (C) Expression of endogenous CCNB1 protein and the active form of the CDK1 protein was determined by western blot using total cell protein extracts obtained from STOX1A siRNA and control treated SH-SY5Y cells. CCNB1 proteins were detected by a specific antibody recognizing total CCNB1 protein. CDK1 proteins were detected using an antibody detecting total CDK1 protein levels and a specific antibody recognizing the active form of CDK1 phosphorylated at threonine 161. An antibody specific for actin was used as a loading control. Westernblot image is a representative of at least 3 independent experiments.

Reduced cell proliferation by STOX1A siRNA knockdown and increased cell proliferation by STOX1A overexpression suggest STOX1A dependent cell cycle regulation. To test this we measured mRNA levels of four major mammalian cell cycle regulatory genes; cyclin A2 (CCNA2, involved in the S to G2 phase and G2 to M phase progression [21]), cyclin B1 (CCNB1, involved in G2 to M phase progression [4]), cyclin C (CCNC, involved in G0 to G1 phase progression [22]) and cyclin E1 (CCNE1, involved in G1 to S phase progression [23]). In stably STOX1A transfected cells we found that CCNA2 and CCNB1 mRNA levels were significantly increased. CCNC mRNA levels were significantly downregulated but no significant differences in mRNA levels were seen for CCNE1 compared to stably MOCK transfected cells (Fig. 3A). STOX1A siRNA knockdown resulted in significantly decreased mRNA levels of CCNA2, CCNB1 and CCNE1 and significantly upregulated CCNC mRNA levels compared to scrambled controls (Fig. 3B). Since CCNB1 is the cyclin involved in mitosis, CCNB1 reduction upon STOX1A knockdown was also confirmed on westernblot. The CCNB1 associated kinase CDK1 showed reduced activity upon reduced CCNB1 protein levels as measured on westernblot using an antibody specifically recognizing the active form of CDK1 (Fig. 3C). In contrast, comparable protein levels of total CDK1 were found (Fig. 3C).

### STOX1A directly regulates expression of CCNB1 thereby enhancing progression into mitosis

**Figure 4:**
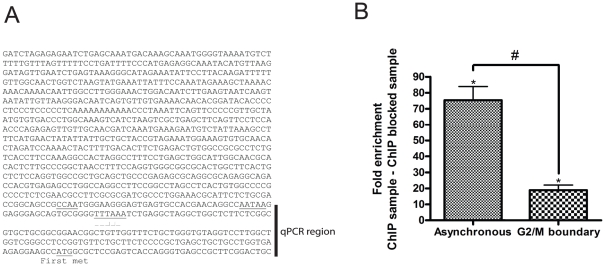
STOX1A binds to the 5′ upstream regulatory region of the CCNB1 gene. (A) The DNA sequence previously characterized as the 5′ upstream regulatory region of the CCNB1 gene [24], [25] indicating the fragment used for quantitative PCR. (B) Chromatin immunoprecipitation was performed with asynchronized STOX1A stably transfected cells in parallel with STOX1A stably transfected cells synchronized at the G2/M phase boundary. Significantly higher enrichment for the 5′ upstream regulatory region of the CCNB1 gene was found in asynchronized cells compared to cells synchronized at the G2/M-phase boundary (left vs right bar). Bars are mean ± SEM, *P*-values were calculated using two-tailed unpaired *t*-test. # indicates *P*<0.001. (B, left bar). Quantitative PCR results show a mean 75 fold (mean ΔΔCt is −6.23) enrichment for the 5′ upstream regulatory region of the CCNB1 gene in STOX1A stimulated ChIP DNA, compared to their negative controls (ChIP sample vs ChIP negative sample) obtained from asynchronized STOX1A stably transfected SH-SY5Y cells. (B, right bar) Results show a mean fold 18 (mean ΔΔCt is −4,18), enrichment for the 5′ upstream regulatory region of the CCNB1 gene in STOX1A stimulated ChIP DNA, compared to their negative controls (ChIP sample vs ChIP negative sample) obtained from STOX1A stably transfected SH-SY5Y cells synchronized at the G2/M-phase boundary. Bars are mean ± SEM. Asterisks indicate *P*<0.05 (one sample t-test with theoretical mean 0).

Given the importance of CCNB1 at the G2/M-phase boundary [6], and our results showing CCNB1 expression to be affected by both knockdown and overexpression of STOX1A we tested if STOX1A could also be directly involved in the regulation of CCNB1 through binding to its promotor region. Therefore we performed chromatin immunoprecipitation (ChIP). The 5′ upstream regulatory region of the CCNB1 gene [24], [25] (Fig. 4A) was tested for enrichment in STOX1A ChIP DNA compared to the negative control in unsynchronized stably STOX1A transfected SH-SY5Y cells. Because STOX1A expression analysis shows a non-overlapping STOX1A immunofluorescence-DNA (DAPI) pattern at specific phases during mitosis we speculated that a direct interaction of STOX1A with the 5′ upstream regulatory region of the CCNB1 gene is like most transcription factors transiently lost during mitosis. Therefore, in parallel, we also tested enrichment of the 5′ upstream regulatory region of the CCNB1 gene [24], [25] in stably STOX1A transfected SH-SY5Y cells arrested at the G2/M-phase boundary. We found significant enrichment for the 5′ upstream regulatory region of the CCNB1 gene [24], [25] in STOX1A ChIP DNA compared to their negative controls in unsynchronized stably STOX1A transfected SH-SY5Y cells and cells synchronized at the G2/M-phase boundary (Fig. 4B). Furthermore, enrichment for the 5′ upstream regulatory region of CCNB1 in the unsynchronized cells (Fig. 4B, left bar) was compared with cells synchronized at the G2/M-phase boundary (Fig. 4B, right bar). As expected, cells that were harvested directly after a G2/M-phase block showed a significantly lower enrichment for the 5′ upstream regulatory region of the CCNB1 gene compared to cells that were growing asynchronously (Fig. 4B).

**Figure 5:**
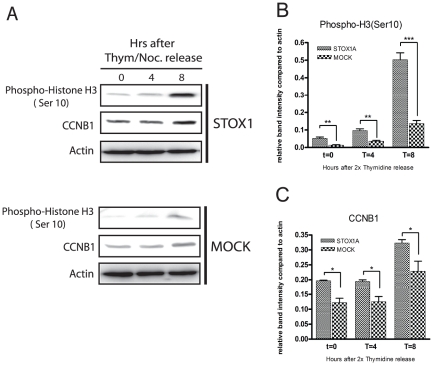
Direct upregulation of CCNB1 effectuates enhanced mitotic entry. (A) Stably transfected STOX1A and MOCK SH-SY5Y cells were synchronized in the S-phase and total cell extracts were prepared from 0, 4 and 8 h after release from the 2× thymidine block. Western blotting was performed using antibodies to Phospho-Histone H3 (Ser 10) and CCNB1. An antibody specific for actin was used as a loading control. Westernblot image is a representative of at least 3 independent experiments. (B) Westernblot (see 5A) quantification of Phospho-H3 (ser 10) was performed using densitometry. The ratio number of obtained band intensities for STOX1A divided by actin was compared to the ratio number of obtained band intensities for MOCK divided by actin for 3 independent experiments. A significant increase for the STOX1A ratio number was found at all time points compared to the MOCK ratio number. (C) Westernblot (see 5A) quantification of CCNB1 was performed using densitometry. The ratio number of obtained band intensities for STOX1A divided by actin was compared to the ratio number of obtained band intensities for MOCK divided by actin for 3 independent experiments. A significant increase for the STOX1A ratio number was found at all time points compared to the MOCK ratio number. *P*-values were calculated using two-tailed unpaired *t*-test, error bars represent ± SEM, * indicate *P*<0.05, ** indicate *P*<0.01, *** indicate *P*<0.001.

To see if the direct regulation of STOX1A on CCNB1 expression would influence transition into the G2/M-phase boundary we synchronized STOX1A and MOCK stably transfected cells in the early S phase with thymidine. 0, 4 and 8 hours after thymidine release CCNB1 protein expression levels were significantly higher in STOX1A compared to MOCK transfected cells (Fig. 5). Furthermore, western blot analysis also showed significantly higher protein expression of the mitosis marker phospho-histone H3 (ser 10) in STOX1A compared to the MOCK transfected cells at these timepoints (Fig. 5).

## Discussion

Here we show, in addition to other members of the forkhead transcription factors, that STOX1A is directly involved in controlling the cell cycle via CCNB1. CCNB1 expression is directly regulated via STOX1A by binding to a region previously characterized as the 5′ upstream regulatory region of the CCNB1 gene [24], [25]. While CCNB1 is known to be a key regulator for mitotic entry [26], the direct up-regulation of CCNB1 by STOX1A let us to speculate if this would have an effect on this phase of the cell cycle. Indeed, in stably STOX1A transfected SH-SY5Y cells that were released from an S-phase block an earlier appearance of the specific mitosis marker PhosH3 (ser10) in parallel with higher CCNB1 protein levels was found. These results show that STOX1A transfected cells progress more rapidly into mitosis compared to control transfected cells. The finding that knockdown of STOX1A causes a decrease in CCNB1 protein levels and that the activity of CDK1 is also dramatically reduced elucidates a role for STOX1A in mitotic entry.

Enrichment of the 5′ upstream regulatory region of the CCNB1 gene in STOX1A transfected SH-SY5Y cells growing asynchronously was significantly higher than in cells synchronized at the G2/M-phase boundary. This implies that during the G2/M phase of the cell cycle the transcriptional potential of STOX1A is temporarily lost. We therefore speculate that STOX1A is necessary for the entry of mitosis but transcriptional activity is transiently lost during mitosis. This is likely happening in metaphase and anaphase. During these phases STOX1A is not bound to DNA. This has also been shown for the forkhead transcription factor FOXK2 [10] and is in general also found for most other transcription factors which temporarily lose their transcriptional potential during mitosis. This is in contrast to FoxI1 which continually remains associated with DNA during mitosis [27]. The translocation of STOX1A away from DNA in these specific phases during mitosis, thereby losing the potential to activate or repress target gene transcription, is possibly necessary for proper mitotic progression or exit.

CCNB1 concentrates at the centrosomes in metaphase during mitosis [17]. While centrosomes serve as reaction centres for several key regulators of the cell cycle machinery [14], [15], and several forkhead transcription factors have been shown to physically interact with the cyclin B1 protein [8]–[10], we hypothesize that STOX1A also interacts with the cyclin B1 protein. Indeed, preliminary data using co-immunoprecipitation suggest this interaction (data not shown). This interaction further suggests that CDK1, the active binding partner of CCNB1, is potentially capable of regulating the transcriptional activity of STOX1A by phosphorylation. Previously, CDK1 has been shown to phosphorylate FOXM1 [8], FOXO1 [9] and FOXK2 [10] thereby influencing their activity.

While we studied the effect of STOX1A on CCNB1 in detail, it is likely that other cell cycle associated proteins are also directly or indirectly differentially affected by STOX1A. Significant changes of CCNA2 and CCNC1 mRNA levels upon STOX1A overexpression and knockdown, and significant downregulation of CCNE1 upon STOX1A knockdown suggest that STOX1A is also involved in regulating other checkpoints of the cell cycle.

As STOX1A is highly expressed in the brain and upregulated in LOAD makes exploration of STOX1A expression in neuronal cells in combination with cell cycle events very interesting; STOX1A might have a role in cell cycle re-entry as observed in AD.

In conclusion, we show for the first time that STOX1A is involved in regulating cell cycle events by binding to CCNB1 thereby regulating mitotic entry.

## Materials and Methods

### Cell culture and transfection

SH-SY5Y human neuroblastoma cells were obtained from the American Type Culture Collection (ATCC, Manassas, VA). All reagents for cell culture were purchased from Invitrogen Life Technologies, Inc. (Burlington, Canada). SH-SY5Y cells were cultured at 37°C in a humidified atmosphere of 5% CO_2_ in Iscove's Modified Dulbecco's Medium (IMDM) supplemented with 10% fetal calf serum and 100 U/ml penicillin, and 100 g/ml streptomycin. Cells were subcultured in medium every 2–3 days following harvesting by trypsinization (HBSS containing 5% trypsin). The ORF (Open Reading Frame) of the STOX1A gene was subcloned into the pF5K-neomycin CMV Flexi vectors according to the manufacturers protocol (Promega) and transfected into SH-SY5Y cells. For transfection the calcium phosphate method was used [28]. Briefly, at the time of transfection, cells were at 70% confluence. By vortexing 2× HEBS (HEPES-buffered saline) with a solution of 2.5 M CaCl_2_ and 20 µg of plasmid DNA a co-precipitate of DNA and CaPO_4_ was allowed to form. After incubation for 30 min at room temperature, the precipitate was added to the cells and the medium was changed after 24 h. For stable transfections SH-SY5Y cells were transfected either with the pF5K-STOX1A or empty pF5K constructs. Selection of positive clones was possible due to the presence of the neomycin resistant gene present in the constructs. Positive clones were maintained in complete IMDB medium supplemented with 800 µg/ml G418 (Roche) until reaching confluence and subcultured every 2–3 days for about 4 weeks. A population of positive clones was harvested for further analysis. As confirmed with qRT-PCR (See below) using TaqMan probes (Applied Biosystems) for STOX1A, 3 stable SH-SY5Y cell-lines with an at least 10 fold STOX1A over-expression compared to mock transfected cells were selected. Additionally, plasmid DNA quantification using qPCR (see below) with primers and probe specific for the constructs backbone showed similar copy numbers between STOX1A and MOCK transfected cells.

Knockdown of STOX1A was performed by transfecting 4 siRNA's against STOX1A and control siRNA (Qiagen) in SH-SY5Y cells with Lipofectamine™ RNAiMAX (Invitrogen) according to the manufactures protocol. Cells were harvested 48 hours post-transfection and RNA was isolated as described in the Quantitative PCR and RT-PCR section.

### Cell proliferation assay *in vitro*

In each well of a 6-well culture plate 1.5×10^5^ untransfected, STOX1A or MOCK stably transfected SH-SY5Y cells were seeded. The untransfected cells were transfected with STOX1A siRNAs (see above). The cell numbers from three wells were counted every day with a time interval of 24 hours for a total of 72 hours. Viable cells were counted on a Countess® Automated Cell Counter (Invitrogen) according to the manufactures instructions. Three independent experiments were performed, and the means were used to depict the growth curve.

### Cell synchronization

Cells were synchronized in an early S-phase by double thymidine treatment. Briefly, ±20% confluent cells were treated with 2 mM thymidine for 16 h. After being released to normal growing medium for 8 h, the cells were treated with 2 mM thymidine for an additional 16 h. Again, after being released to normal growing medium, cells were harvested or analysed in time intervals of 2 hours for a total of 8 hours.

For cells to be synchronized at the G2/M-phase boundary, cells were treated with a thymidine/nocadazole block. Briefly, ±40% confluent cells were treated with 2 mM thymidine for 24 h. After being released to normal growing medium for 3 h, the cells were treated with 100 ng/ml nocodazole for 12 h and the cells were arrested at the G2/M-phase boundary.

### Chromatin immunoprecipitation

Stably transfected STOX1A cells (asynchronous or G2/M-phase boundary arrested) were treated with formaldehyde to create protein-DNA crosslinks. Cytoplasmic lysis was performed to reduce competition of cytoplasmic STOX1A-Halotag proteins against nuclear STOX1A-Halotag proteins with the Halotag resin. Nuclear lysate was subsequently fragmented by sonication. The nuclear lysates were split into two equal parts of which one was treated with Halotag blocking ligand to function as a negative control. Both the samples and controls were treated using Halotag resin according to the Halo-ChIP system protocol in the presence of proteinase inhibitors. After reversal of crosslinks the DNA was purified using the Qiaquick PCR purification kit (Qiagen).

### Quantitative PCR and RT-PCR

Standard quantitative PCR was performed on an ABI7300 (Applied Biosystems) using a probe and primers specific for a fragment in the 949-bp region previously characterized as the 5′ upstream regulatory region of the CCNB1 gene ^24^. Probe and primer characteristics were: CCNB1_foward 5′ GTGCGGGGTTTAAATCTGAG 3′, CCNB1_reversed 5′ CATGGCTTCCTCTTCACCAG 3′ and 5′-FAM 3′-TAMRA labeled probe: 5′ TGTTCTGCTTCTCCCCGCTG 3′. Reactions were performed in the presence of 1 M betaine and ROX reference dye, and corrected for input using the non-intron-spanning Glyceraldehyde 3-phosphate dehydrogenase (GAPDH) gene expression assay (Applied Biosystems). Input ChIP DNA was obtained from at least four independent ChIP experiments.

RNA isolation from transfected cells was performed using the RNeasy kit (Qiagen) including on-column DNase treatment. Quantitative RT-PCR using gene expression assays (Applied Biosystems) for CCNA2, CCNB1, CCNC, CCNE1 and STOX1A were performed on an ABI7300. In addition, transfection effiencies were corrected by plasmid DNA quantification using the pF5 CMV-neo Flexi® Vector backbone present in all plasmids (pF5 forward: 5′-GCTTCGAGCAGACATGATAAG-3′, pF5 reversed: 5′-AGCAATAGCATCACAAA TTTCA-3′, 5′-FAM 3′-TAMRA labeled pF5 probe: 5′-TGGACAAACCACAACT AGAATGCAGT-3′). Data were obtained from at least four transfections from which each RNA sample was measured in triplicate.

### Western blot

Protein lysates from transfected cells were obtained by directly scraping cells into Loading Buffer including *β*-mercaptoethanol. Lysates were separated by SDS-polyacrylamide gel electrophoresis, and electroblotted onto a PVDF-membrane. An antibody recognizing endogenous STOX1 (Sigma), Phospho-cdc2 Thr161 (cell signalling) or Phospho-Histone H3 (cell signalling) was used in combination with goat anti-rabbit horseradish peroxidase-conjugated secondary antibody (DAKO). An antibody recognizing CCNB1 (Millipore) and total cdc2 (clone POH1, cell signalling) were used in combination with goat anti-mouse IgG horseradish peroxidase-conjugated secondary antibody (DAKO). Protein bands were detected by an enhanced-chemiluminescence assay (GE Healthcare) on a LAS3000.

### Immunofluorescence

For immunofluorescence, stably transfected SH-SY5Y cells were grown on glass coverslips. Coverslips were fixed in 4% (PFA) paraformaldehyde for 15 min at room temperature. After fixation, coverslips were rinsed in PBS, 0.1% Triton X-100 and incubated with 1% Triton X-100 in PBS for 15 min at room temperature for permeabilization. Coverslips were washed in wash buffer (PBS, 0.1% Triton X-100, 2% BSA (Bovine serum albumin)) blocked with PBS, 0.1% Triton X-100, 5% BSA for 1 h and incubated with anti-Halotag (Promega) antibody at 4°C overnight. After washing with wash buffer, cells were incubated for 1 hour with anti-rabbit secondary antibodies conjugated with Alexa Fluor 568 (Invitrogen), washed with washing buffer and mounted with vectashield mounting solution containing DAPI for DNA counterstaining (Vector Laboratories).

### Data analysis

For Real-time PCR data, a threshold cycle number, Ct, was measured as the PCR cycle at which the amount of amplified target reaches the threshold value. Quantification was determined by the 2^−ΔΔCt^ method as described in Applied Biosystems “Guide to Performing Relative Quantitation of Gene Expression Using Real-Time Quantitative PCR”, Section VII, Relative Quantitation of Gene Expression Experimental Design and Analysis http://www3.appliedbiosystems.com/cms/groups/mcb_support/documents/generaldocuments/cms_042380.pdf). Statistical analysis of the obtained data was carried out with the GraphPad Prism program.
